# Uptake of Task-Strengthening Strategy for Hypertension (TASSH) control within Community-Based Health Planning Services in Ghana: study protocol for a cluster randomized controlled trial

**DOI:** 10.1186/s13063-020-04667-7

**Published:** 2020-10-02

**Authors:** Kwaku Poku Asante, Juliet Iwelunmor, Kingsley Apusiga, Joyce Gyamfi, Solomon Nyame, Kezia Gladys Amaning Adjei, Angela Aifah, Kwame Adjei, Deborah Onakomaiya, William F. Chaplin, Gbenga Ogedegbe, Jacob Plange-Rhule

**Affiliations:** 1 Kintampo Health Research Centre, Ghana Health Service, P.O. Box 200, Kintampo, Ghana; 2 Department of Behavioral Science and Health Education, College for Public Health and Social Justice, Saint Louis University, St. Louis, MO USA; 3 Department of Physiology, School of Medical Science, Kwame Nkrumah University of Science & Technology, Kumasi, Ghana; 4 Department of Population Health, NYU Langone Health, New York, NY USA; 5 Department of Psychology, St. John’s University, Queens, NY USA

**Keywords:** Task strengthening, Hypertension control, Practice facilitation, Implementation science, Community-Based Health Planning Services, Ghana, Sub-Saharan Africa

## Abstract

**Background:**

Physician shortage is a major barrier to hypertension (HTN) control in Ghana, with only one physician to 10,000 patients in 2015, thus limiting its capacity for HTN control at the primary care level such as the Community Health Planning and Services (CHPS) compounds, where most Ghanaians receive care. A Task-Shifting Strategy for HTN control (TASSH) based on the WHO Cardiovascular (CV) Risk Package is an evidence-based strategy for mitigating provider- and systems-level barriers to optimal HTN control. Despite its effectiveness, TASSH *remains untested* in CHPS zones. Additionally, primary care practices in low- and middle-income countries (LMICs) lack resources and expertise needed to coordinate multilevel system changes without assistance. The proposed study will evaluate the effectiveness of practice facilitation (PF) as a quality improvement strategy for implementing TASSH within CHPS zones in Ghana.

**Methods:**

Guided by the Consolidated Framework for Implementation Research and the Reach, Effectiveness, Adoption, Implementation, and Maintenance framework, we will evaluate, in a hybrid clinical effectiveness-implementation design, the effect of PF on the uptake of an evidence-based TASSH, among 700 adults who present to 70 CHPS zones with uncontrolled HTN. Components of the PF strategy include (a) an advisory board that provides leadership support for implementing the intervention within the CHPS zones and (b) trained task-strengthening facilitators (TSFs) who serve as practice coaches to provide training, and performance feedback to community health officers (CHOs) who will deliver TASSH at the CHPS zones. For this purpose, the TSFs are trained to identify, counsel, and refer adults with uncontrolled HTN to community health centers in Bono East Region of Ghana.

**Discussion:**

Uptake of community-based evidence-supported interventions for hypertension control in Ghana is urgently needed to address the CVD epidemic and its associated morbidity, mortality, and societal costs. Findings from this study will provide policymakers and other stakeholders the “how to do it” empirical literature on the uptake of evidence-based task-strengthening interventions for HTN control in Ghana and will serve as a model for similar action in other low, middle-income countries.

**Trial registration:**

ClinicalTrials.gov, NCT03490695. Registered on 6 April 2018.

**Protocol version and date:**

Version 1, date: 21 August, 2019.

## Background

Shortage of healthcare workers is a major barrier to hypertension (HTN) control in Ghana, where in 2017 the density of doctors, nurses, and midwives was 2.65 per 1000 population [1, 2]. This acute shortage of physicians limits Ghana’s capacity to control HTN in adults at the basic primary care level such as the Community-Based Health Planning and Services (CHPS) zones, where majority of Ghanaians receive their care [3]. The shortage of health workers is partly due to brain drain of health workers to high-income countries for better standard of living, higher salaries, and stable political conditions [1, 4, 5]. There is thus an urgent need for strategies that help reduce the burden of NCDs in Ghana, particularly community-based strategies targeted at the rational use of available resources [6, 7]. One such approach is a task-shifting strategy, defined as the rational distribution of primary care duties from physicians to non-physician healthcare providers [8–12].

Task shifting is useful in low-resource settings, such as SSA countries, who face acute healthcare human resource crisis [8–12]. In order to maximize the efficient use of resources, healthcare tasks are shifted from higher-trained health workers to less-trained health workers [8, 9]. In this context, shifting the tasks of CV risk assessment and management of uncomplicated CV risk factors, such as HTN from physicians to non-physician healthcare providers (NPHCPs) such as community health workers and nurses, is a viable and cost-effective strategy. Growing evidence suggests that patients with HTN can be cared for by NPHCPs, who provide adequate counseling of healthy lifestyle to patients almost as often as physicians [8, 13].

We recently demonstrated, in a cluster randomized control trial in 32 district hospitals and community health centers in Ghana, that an evidence-based Task-Shifting Strategy for HTN control (TASSH) based on the WHO Cardiovascular Risk Package, delivered by community health nurses (CHNs), led to 1.34 times greater systolic blood pressure reduction than provision of health insurance [13]. Although TASSH led to a significant reduction in systolic BP, its widespread implementation and dissemination within the Ghana’s Community-Based Health Planning and Services (CHPS) zones have not been evaluated. CHPS zones are unique settings for implementation of TASSH because of their use of community health officers (CHOs) who deliver primary care services [3]. At the hub of this primary care system is the widespread implementation of the CHPS program, which represent the “ground level” of Ghana’s health system, and provide basic primary care services to people in their communities, through community engagement in the planning and delivery of services [3]. CHPS utilizes CHOs living in a defined community to offer limited curative and preventive healthcare services to the population [3]. The use of CHPS as a platform for healthcare delivery in every community in Ghana [3] allows us to evaluate the adoption and maintenance of TASSH in community settings.

This study provides a unique opportunity for us to fill this research-to-practice gap by evaluating the effectiveness of a practical and replicable strategy for implementing TASSH among adults who present to CHPS zones with uncontrolled HTN*.* Systematic reviews show that effective implementation strategies of evidence-based interventions are typically multilevel, and are tailored to the practice context [14, 15]. However, primary care practices in low- and middle-income countries (LMICs) lack resources and expertise needed to coordinate multilevel system changes without assistance [16]. One implementation strategy that may effectively overcome this barrier is practice facilitation (PF) via provision of external expertise on practice redesign, and a tailored approach to implementing guideline-concordant care [16, 17]. Although PF is well studied in high-income countries [18–20], its role remains untested in LMICs.

### Objectives

The study has four specific objectives:

- *Aim 1*: Identify practice capacity for the adoption of TASSH in CHPS zones and develop a culturally tailored PF strategy using qualitative methods.
- *Aim 2a*: Evaluate in a cluster randomized controlled trial (RCT) the effect of PF versus usual care (UC) on the adoption of TASSH across 70 CHPS zones at 12 months post-randomization.
- *Aim 2b*: Compare in a cluster RCT design the clinical effect of PF versus UC on systolic blood pressure (BP) reduction among 700 adults with uncontrolled HTN at 12 months post-randomization.
- *Aim 3*: Evaluate the sustainability of TASSH implementation across the CHPS zones at 24 months post-randomization (1 year after completion of the trial).
- *Aim 4*: Evaluate the mediators of the adoption of TASSH across the CHPS zones at 12 months post-randomization.

## Methods

### Trial design

Using a hybrid clinical effectiveness-implementation design [21], we will conduct this study in 3 sequential phases: (1) The first is a pre-implementation phase, based on the Consolidated Framework for Implementation Research (CFIR) [22] to assess inner setting variables and provider characteristics likely to influence the adoption of TASSH within the CHPS zones. This information is used to develop and user-test a culturally tailored PF strategy to implement TASSH. (2) Next is an implementation phase, which evaluates in a cluster RCT the effect of the PF strategy versus usual care on the adoption of TASSH as well as clinical effectiveness on systolic BP reduction at 12 months post-randomization across the CHPS zones. We will also evaluate the mediators of the adoption of TASSH in the CHPS compounds. (3) Another is a post-implementation phase which evaluates the sustainability of TASSH implementation across participating CHPS zones at 24 months (1 year after completion of the trial). Measures for the implementation and the post-implementation phases are guided by CFIR and the RE-AIM framework [22–25].

### Study setting and participant characteristics

The study is currently ongoing in 70 of the 97 CHPS zones in three contiguous districts within the Bono East Region (Kintampo North, Kintampo South District, and Nkoranza North District) of Ghana. The study area is predominantly rural and mostly farming communities [26, 27]. About 40% of the adult population have no education. Access to healthcare is mainly at the community-level healthcare which is limited. About 50% of the population have valid health insurance cards that enable them to access basic healthcare under the National Health Insurance Scheme. In 2016, 2555 community members aged ≥ 18 years (mean age of 43 years, SD 17; 60.5% female) participated in a cross-sectional health survey, which documents the prevalence of hypertension as 28.1% (95% CI 26.3–29.8), with pre-hypertension prevalence of 42.5% [28]. Approximately 45.9% (95% CI 42.2–49.6) of hypertensive respondents were aware of their diagnosis; of those aware, 41.3 (95% CI 36.1–46.8) sought medical treatment and had their hypertension controlled, while 58.7% (95% CI 53.2–63.9) did not seek any medical treatment. Tobacco use in the area is about 3%, and the prevalence rate of obesity among the adult population (> 40 years) is 11% [28].

### Sites and participant recruitment

Participants will be recruited from eligible CHPS zones that (1) have at least 2 community health workers (CHWs) employed in the CHPS program and (2) are certified as a National Health Insurance Scheme provider. A total of 70 CHPS zones will be recruited into this trial.

Patients for the study are identified from the CHPS zones by the CHWs during provision of their routine services and visits with families or through of the conduct of health fairs in participating CHPS zones. Each zone is expected to recruit consent 10 consecutive patients who meet the eligibility criteria below. Study coordinators will obtain individual consent from eligible patients before they can be enrolled in the study. For patients recruited to the CHPS zones, once eligibility is confirmed, all patients regardless of their group assignment will have a total of four study visits: baseline, 6 months, 12 months, and 24 months post-randomization. The structure of each study visit and the respective measures will be the same for each group. A total 70 CHPS compounds and 700 patients will be enrolled in this study with approximately 10 patients per CHPS compound. Patients may withdraw from or refuse to participate in the trial at any point, and it will not affect the clinical care they receive.

### Inclusion criteria

#### CHPS zones

Eligible CHPS zones must have at least 2 community health officers (CHOs) employed in the CHPS program and be a certified National Health Insurance Scheme provider.

#### Patients

Adults who (a) are patients registered to receive care at CHPS zone, (b) are aged 40 years and older, (c) have average BP (140–179/90–100 mmHg) from two separate visits to the CHPS zone at least 1 week apart, and (d) have the ability to provide informed consent were included.

### Exclusion criteria

CHPS zones that are not a certified National Health Insurance Scheme provider will be excluded.

Patients will be excluded if they meet the following g criteria: (a) those with previous diagnosis of diabetes, stroke, heart failure, or chronic kidney disease; (b) BP > 180/100 mmHg; (c) positive urine dipstick for protein; (d) pregnancy; and (e) inability to provide informed consent. Patients with history of stroke, heart failure, diabetes, angina, claudication, and BP > 180/100 mmHg will be referred to the district hospitals.

### Ethical approval and trial protocol

Ethical approval for this study was obtained from the Kintampo Health Research Centre Institutional Ethics Committee; Kwame Nkrumah University of Science and Technology Committee on Human Research, Publication, and Ethics; and the Ghana Health Service Ethics Review Committee.

### Randomization and blinding

Randomization will occur at the level of the CHPS zones. Each CHPS zone will be randomly assigned to one of the two arms: Group A will receive the practice facilitation strategy herein referred to as a task-strengthening facilitation (TSF) strategy for 12 months. Assessment of adoption ratings on TASSH will be made at 12 months post-randomization, and sustainability will be assessed at 24 months post-randomization. Group B will receive usual care practices (screen, counsel, and refer patients without practice facilitation) at CHPS zones [27]. Adoption ratings of TASSH will be assessed for group B at 12 months post-randomization, and sustainability assessment will occur at 24 months post-randomization. The sequence of randomization will be generated by study statistician and kept in sealed opaque envelopes away from the study sites in accordance with the CONSORT guidelines [29]. Sites will be informed of their randomization group by telephone and email.

#### Masking and blinding

Due to the nature of the intervention, it is impossible to blind the patients, the community health officers, and the study coordinators to the group assignment of each intervention. However, the salient dimension that the study coordinator could possibly affect is the BP measurements. To mitigate this bias, we will use an automated BP device, which prevents the study coordinator from influencing in any manner the BP readings. We anticipate that this trial will not pose any direct harm to the participants as no treatment/medication/supplement will be administered by the trial to participants. Participants will be referred to the nearest district hospital for treatment of any conditions they may develop during the period of this trial. Enrolling in this trial will not prohibit study participants from accessing medical care for any condition they may have.

**Fig. 1:**
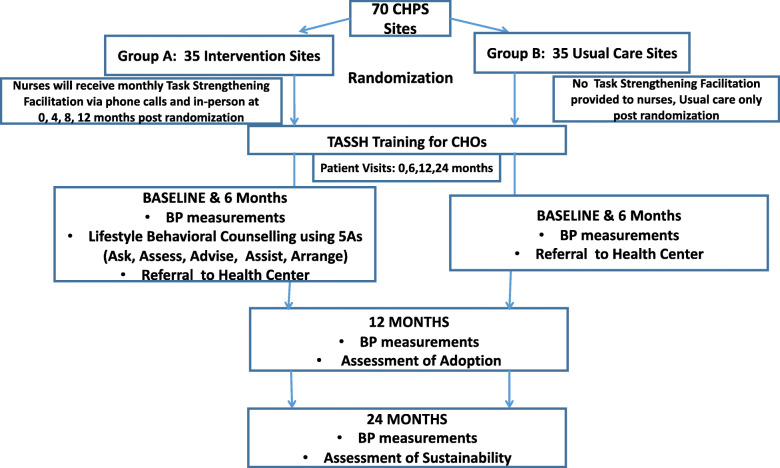
Trial implementation design (parallel)

**Fig. 2:**
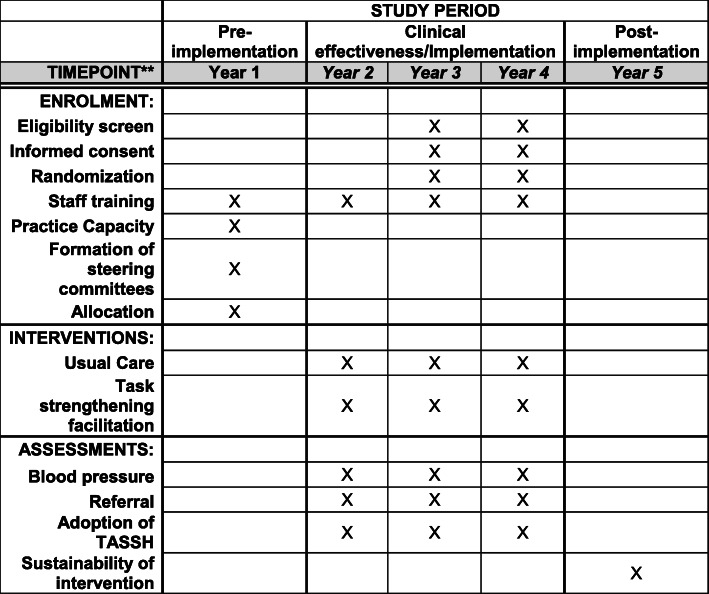
TASSH SPIRIT figure

The trial flow chart is shown in Fig. 1, and the SPIRIT figure is shown in Fig. 2. The SPIRIT checklist is provided in Additional file 1. As shown in Fig. 1, all CHPS zones enrolled will be randomly assigned to either group A, the intervention group (*N* = 35), or group B, the control group (*N* = 35). A total of 70 CHPS compounds and 700 patients will be enrolled in this study with approximately 10 patients per CHPS compound. We considered randomizing patients, but the likelihood of contamination represents a threat to internal validity; thus, patients will be nested within the CHPS compounds. CHPS zones randomized to group A will receive the task-strengthening facilitation (TSF) intervention, as described in the “Intervention conditions” section below. Group B will receive health information based on the TASSH protocol. The only difference is that CHPS zones in the usual care group will not receive practice facilitation. Approximately two CHOs will be trained per CHPS compound, providing a sample of 140 CHOs overall and 70 CHOs per condition. Also shown in Fig. 1 is the facilitation schedule which will occur at months 3, 6, and 9 of the intervention periods and the assessment points in each group.

### Intervention conditions

Participants will be assigned to either the practice facilitation group (group A) or the usual care group (group B).

#### Practice facilitation (group A)

Patients belonging to the intervention group will go through the same process as those of the usual care group. The CHOs in this group will receive the task-strengthening facilitation delivered by the task-strengthening facilitators. The practice facilitation intervention (intervention group) has three components:

i. Training of TSFs on *Engaging*, *Enhancing*, and *Evaluating* their tasks at the CHPS zones. The TSFs will carry out this function via onsite and remote supportive supervision of the CHOs. The basis of the strategy is to *Engage* the CHOs (via monthly phone calls to address barriers that the CHOs may have in performing their duties) and *Enhance* the CHOs (via onsite visits quarterly to observe and supervise them in their duties) and use of learning communities. Study staff will certify and re-certify the TSFs as trainers yearly using a train-the-trainer model. This will be accomplished via a training module consisting of the following: (1) Training on BP measurement and how to identify, screen and refer patients to the health centers for treatment. (2) Training on counseling strategies for engaging CHOs, enhancing them, and evaluating their tasks. The TSFs will be trained to map out the list of tasks that the CHOs will perform, categorize the list of tasks into three groups (screening for diagnosis of HTN, counseling, and referral), and create scenarios and examples of how to use the EEEs around each task. The scenarios will be in Twi language and at elementary school level. (3) Training on use of the online learning communities such as WhatsApp to engage the CHOs. (4) Training on use of remote phone calls and onsite supervision of the CHOs. The TSFs will be trained by study staff to communicate with the CHOs remotely via telephone calls and via onsite visits. The phone calls will occur once a week for at least an hour in duration, while the onsite visits will occur every 4 months for duration of 1 day. CHOs will be trained to develop a list of participants contacted, counseled, and referred using an electronic monitoring system. They will be trained to develop a checklist for the phone calls and another for the site visits.
ii. Training the CHOs on identifying, counseling, and referring (ICR) of adults with HTN to the health centers using the 5 A’s counseling strategy (Ask, Assess, Advise, Assist, and Arrange) [30]. Similarly, study staff will create a training module for the CHOs. Components of the module are as follows: training on BP measurement and training on how to identify, counsel, and refer patients with HTN to the community health centers for treatment. This counseling strategy will use the 5 A’s (Ask, Assess, Advise, Assist, and Arrange) [30]. Duration of the training of the CHOs and TSFs will be three full days. The initial training will be supplemented by booster trainings of the same duration every 3 months in order to minimize drift. In order to guarantee sustainability, study staff in Ghana will be trained to deliver this training to CHOs every 6 months
iii. Creation of a community learning environment to support learning opportunities for the CHOs and TSFs. Study staff will develop information that will be shared on the WhatsApp group with the CHOs and TSFs. Study staff will create a series of messages that will be sent via WhatsApp as a structured blast to CHOs and TSFs. Every 2 weeks, a short message about a topic on HTN will go out to both groups. Similarly, there will be an *Ask the Expert Forum*. These messages will include information on BP measurement techniques, counseling skills, and sharing of hypertension education material. Other information will include tips on identification, counseling, and referral of patients to health centers.

##### Engagement of the community to create a community urgency and persistency of the importance of HTN screening and referral for adults

This component of the intervention will be conducted by the Local Steering Committee that comprises the leadership of the regional health directorate, public health nurses, director of surveillance, coordinator for the community engagement committee, data manager, and the regional NCD resource person. This committee is already convened as part of the Ghana Health Services and will coordinate the community engagement activities via health fairs, visits to health centers, and other events organized by the Regional Health Directorate of the Brong-Ahafo Region where the participating CHPS zones are located.

#### Usual care (group B)

CHOs based at group B facilities will be trained on the 5 A’s and referral of the participants to the health center. However, they will not receive practice facilitation from the TSFs. Participants attending CHPS zones randomized to group B will receive standard care offered by that CHPS facility.

### Study outcomes

*Primary outcome* is the rate of adoption of TASSH at the CHPS zones at 12 months. This will be based on a composite measure of three adoption ratings (i.e., number of newly detected hypertensive patients identified by the CHOs, proportion of patients receiving lifestyle counseling, and referred for further management by the CHOs). The primary outcome will be assessed at 12 months post-randomization by the following measures:

1. *Rate of newly detected hypertensive patients*: Of the number screened by the CHOs, how many were identified as hypertensive (BP > 140/90 mmHg)?
2. *Proportion of patients receiving lifestyle counseling*: Of the number identified by each CHO as hypertensive, how many received lifestyle counseling?
3. *Proportion of eligible patients that were referred to community health centers for further management*: Of the number identified by each CHO as hypertensive, how many were referred to the community health center for further management? In order to assess these measures, the CHOs will complete a questionnaire inquiring about newly diagnosed hypertension and behavioral counseling practices; the retention and referral rates for patient follow-up at each study visit will be monitored. CHOs who have increased rates for all three metrics will be identified as having adopted the TASSH intervention.

*Secondary outcomes* are as follows:

- Mean reduction in patient systolic blood pressure (BP) at 12 months post-randomization.
- Mediators (implementation climate, leadership support, organizational capacity, and provider-level characteristics) of the adoption of TASSH across the CHPS zones at 12 and 24 months will be assessed.
- Rate of adoption of TASSH at the participating CHPS zones at 24 months post-randomization (sustainability).

### Assessment of outcome measures

The study measures are described below.

#### Blood pressure measurements

The systolic BP reduction in patients will assessed as follows as mean change in systolic BP from baseline to 12 months. BP readings will be assessed with validated automated BP device as we did in our previous trial [10]. A total of three readings will be taken by trained study coordinators using an automated BP monitor with the patient seated comfortably for 5 min prior to the measurements, following the AHA guidelines [31]. The same procedure will be followed at 6- and 12-month study visits. The average of the 3 BP readings will be used as the measure for each study visit. Uncontrolled BP is defined as average clinic SBP ≥ 140 mmHg or DBP ≥ 90 mmHg following JNC-7 guidelines [31].

#### Mediators of TASSH uptake

The following measures will be used to assess the potential mediators of the adoption of TASSH across the CHPS zones at the systems, organizational, and provider levels.

##### Systems-level and organizational-level measures

*Organizational Capacity for Change* (*OCC*) [32] is a 32-item multidimensional scale that evaluates an organization’s capacity to upgrade or revise existing organizational competencies, while cultivating new competencies that enable the organization to survive and prosper. It comprises different aspects of leadership, employee behavior, and an organizational infrastructure supporting organizational change. Items are rated on a 5-point Likert scale. OCC has a Cronbach *α* of 0.87.

*Implementation Leadership* will be assessed with the Implementation Leadership Scale (ILS) [33] which has excellent reliability, convergent, and discriminant validity. The ILS is a brief 12-item measure with four subscales: Proactive Leadership (*α* = .95), Knowledgeable Leadership (*α* = .96), Supportive Leadership (*α* = .95), and Perseverant Leadership (*α* = .96) and a total score (*α* = .98) [33].

*Implementation Climate* will be assessed with the Implementation Climate Scale (ICS) [34] that measures shared perceptions of the policies, practices, procedures, and behaviors that are expected, supported, and rewarded to facilitate effective evidence-based practice (EBP) implementation. It has an overall Cronbach’s alpha of .91 (18 items, 3 items on each subscale). The six subscales of EBP Implementation Climate are as follows: Focus on EBP (*α* = .91), Educational Support for EBP (*α* = .84), Recognition for EBP (*α* = .88), Rewards for EBP (*α* = .81), Selection for EBP (*α* = .89), and Selection for Openness (*α* = .91) [34].

*Organizational Culture domain (i.e., Proficient Culture) of the Organizational Social Context Scale* [35] is a 15-item proficiency subscale that will be used to evaluate the practice capacity proficiency level at participating CHPS compounds. Proficient Organizational Cultures [36] are those characterized by shared norms and expectations that the CHOs place the well-being of each patient first, are skilled service providers, and have up-to-date knowledge of the TASSH protocol. Items are completed using a 5-point rating scale ranging from 1 (never) to 5 (always) with measures such as responsiveness (e.g., “members of my organizational unit are expected to be responsive to the needs of each patient”) and competence (e.g., “members of my organizational unit are expected to have up-to-date knowledge”). Alpha reliability for the proficient culture scale is .89 [35, 36].

##### Provider-level measures

*Evidence-Based Practice Attitude Scale* (*EBPAS*) [37, 38] is a 15-item measure with four subscales that assess attitudes toward adoption of EBP as a function of perceived appeal of EBP, requirements to use EBP, provider openness, and perceived divergence between EBP and usual care. EBPAS total scores (*α* = .76) represent global attitudes toward adoption of EBP. EBPAS responses are scored on a 5-point scale (*0 = Not at all*, *4 = To a Very Great Extent*), and scores are associated with provider-level attributes and organizational characteristics [37, 38].

*Organizational Change Recipients’ Beliefs Scale* [39] is a 24-item tool that evaluates the following: (a) the degree of buy-in among change recipients (the CHOs), (b) deficiencies in specific beliefs that can adversely impact the success of organizational change, and (c) planning and executing actions to enhance buy-in among organizational change recipients. Items are scored on a 5-point Likert scale. The Cronbach *α* ranges from .70 to .94 [39].

#### Sample size and power estimates

The proposed design is a cluster randomized design to compare PF versus usual care. The primary outcome is a composite measure of adoption ratings to assess the degree to which the TASSH protocol is implemented at the CHPS compounds. The secondary outcomes are the difference in the change in systolic BP between patients at the CHPS sites randomized to PF versus those at the usual care CHPS sites. The unit of randomization is the CHPS compound, and the total sample size is the average number of CHOs at the CHPS sites who will provide the adoption ratings (primary outcome) at each site × the number of CHPS compounds and the average number of patients whose systolic BP will be assessed (secondary outcome) enrolled at each CHPS compound × the number of CHPS compounds. In order to estimate the number of CHPS compounds and the average number of CHOs and the average number of patients needed to have power of .80 for a hypothesis test with a type 1 error rate (alpha) of .05, we have assumed a moderate standardized effect size (*d*) of .50 of the difference in change in adoption ratings between the CHOs in the CHPS sites in the UC group versus those in the CHPS sites in the PF group from baseline to 12 months. We note that Baskerville et al. reported in a systematic review of practice facilitation in primary care settings an average effect size of *d* = .56 [15]. We also calculated the sample size for an assumed degree of dependency (intra-cluster correlation—ICC) of the CHOs’ data within CHPS compounds of .10. Thus, for an ICC of .10 and 2 CHOs providing the adoption ratings, our target number of CHPS compounds to recruit is 70. As such, a total of 140 CHOs will participate in this study. For the secondary outcome of patient change in systolic BP, we will have substantially greater power than .80 under the same effect size (*d* = .50) and ICC assumptions as we anticipate enrolling an average of 10 patients/compound. So, with 70 compounds, we would enroll 700 patients. Aim 3 concerns the correlation relationship between different mechanisms of change and the change in adoption ratings between baseline and 12 months. For this analysis, we will have the entire sample of 70 compounds and 140 nurses. With this sample and the same assumptions that we made for aim 2a and aim 2b, we will have power of .80 to detect a moderate correlation of .30. Finally, aim 4 concerns detecting a quadratic pattern consistent with sustainability across the baseline and 12- and 24-month time points. Again, the entire sample will be available for this analysis. The standardized effect size for the quadratic effect will depend considerably on the exact form of the quadratic pattern. The largest effect size would occur when the adoption is not sustained; that is when the adoption ratings revert to their baseline values after PF has ended. With the proposed sample size, we will have power greater than .90 to determine if this unwelcome event occurs.

### Statistical methods for primary and secondary outcomes

#### Analysis for aim 1: Identify practice capacity for the adoption of TASSH in CHPS zones and develop a culturally tailored PF strategy using qualitative methods

The semi-structured interviews and user-testing interviews will be transcribed and entered into NVivo, version 11, for data analysis. We will use the framework approach to qualitative data analysis [40], a five-step process that involves the following: (i) familiarization (a process during which the researcher becomes immersed in the details of multiple sources of data to gain a general understanding of the content and to document initial impressions), (ii) developing a theoretical framework (a process by which the researcher identifies emergent themes in the multiple sources of data using existing theories as a guide [40]; these themes will be continually refined and compared to each other), (iii) indexing (during which the researcher becomes further immersed in the data in order to refine identified themes and subthemes), (iv) summarizing data in an analytical framework (during which the researcher reduces materials into understandable, but brief summaries of what was said by stakeholders), and (v) data synthesis and interpretation (which allows for comparison of themes and subthemes against original transcripts, field notes, and audio recordings to ensure appropriate context [40]). Following the framework approach, as described above, the data will be independently coded by the two-three research members to reduce the potential for bias. Inter-rater reliability will be determined based on a subset of the data, for example, the interviews, and will be repeated until satisfactory agreement among raters is achieved (i.e., 80% of coded data). Discrepancies in coded data will be resolved by consensus. After systematically reading all transcripts, they will be coded into concepts reflecting the aim of this phase. For example, responses will be coded according to provider-level factors (e.g., knowledge of TASSH toolkit) and issues related to practice capacity with using the TASSH toolkit in CHPS (e.g., logistics/resources involved). Established procedures to enhance the trustworthiness of our analysis will be used [41], including analyses of codes that do not fit our coding scheme, development of an audit trail documenting analytical decisions, and member-checking presentations to the Steering Committee. The identified concepts will then be grouped into categories, and themes uniting the categories will be determined. A detailed analysis of the interviews should generate a conceptual model that elucidates barriers to the uptake of the TASSH toolkit within the CHPS compounds and modified PF strategy tailored to the CHPS context to overcome these challenges.

#### Analysis for aim 2a: The adoption of TASSH will be higher in the CHPS compounds randomized to the PF strategy than those in usual care at 12 months post-randomization

This analysis will be accomplished with a multilevel mixed model using an unstructured covariance matrix across two time points, baseline and 12 months. The analysis will have one within-person factor—time (baseline and 12 months coded naturally as months 0 and 12)—and one primary between-patient factor (randomization group dummy coded as 0 = UC and 1 = PF). Fixed effects will be specified for time, randomization group, and their interaction effect (group by time). The outcome measure will be a composite index for adoption of TASSH (defined as rate of identification of HTN, rate of lifestyle counseling for patients with HTN, and medication referral for patients with HTN). Additionally, the CHOs will be nested within compounds creating a 3-level analytic model (observations nested within CHOs nested within compounds). Random effects will be specified for CHPS and for CHO, adjusting for the clustering of measures within CHO and CHO within CHPS. Multilevel modeling software (SAS, version 9, PROC MIXED) will be used to compute full information maximum likelihood (FIML) estimates of the model parameters. The PROC MIXED procedure will use an error structure that allows for the possibility of group differences in (a) the error variances at 12 months and (b) the serial correlations of the baseline with the 12-month outcomes. The primary test is the group × time interaction, and the resulting *F* test will provide the primary “intent to treat” test of the hypothesis. If this is statistically significant at the two-tailed *α* = .05 level, for ease of interpretation, we will estimate and report the magnitude of the treatment effect, with 95% CI for adoption. Ideally, the randomization of participants to treatment groups will obviate the need for any covariates in the analysis. However, in the event that baseline differences between the patients in each group on the outcomes, demographic, or secondary measures are found, those variables will be included as covariates in the model (including their interactions with time).

#### Analysis for aim 2b: Mean systolic BP will be lower among patients in the CHPS compounds randomized to the PF strategy than those in usual care at 12 months post-randomization

This analysis will be accomplished using a similar analytic strategy as described for the quantitative analysis for aim 2a. However, in this analysis, the measurements will be taken at the level of the patient rather than the CHO. We will specify a generalized linear multilevel mixed model with fixed effects for time, intervention, and intervention by time. Random effects will be specified for CHPS, CHO, and patient, accounting for measurements clustered within patient, patients clustered within CHO, and CHOs clustered within CHPS. SBP will be measured as a continuous variable in mmHg at baseline and 12 months for all patients. Multilevel modeling software (SAS, version 9, PROC MIXED) will be used to compute full information maximum likelihood (FIML) estimates of the model parameters for the fixed and random effects.

#### Analysis for aim 3: Sustainability of TASSH will be higher at the CHPS zones randomized to PF than those in CHPS zones randomized to usual care at 24 months post-randomization

Sustainability will be defined as adoption of TASSH at 24 months (12 months following the completion of the intervention). As in aim 2a, this analysis will be conducted at the level of the CHO. The analysis will be accomplished with a multilevel mixed model using an unstructured covariance matrix across two time points, baseline and 24 months post-randomization. Fixed effects will be specified for time, group, and group by time. Random effects will be specified for CHO and CHPS to account for the clustering of measurements.

#### Analysis for aim 4: Inner setting variables (implementation climate, leadership support, organizational capacity) will mediate the effect of PF on adoption of TASSH at 12 months following randomization and sustainability at 24 months following randomization

In this analysis, we will evaluate the mediators of adoption and sustainability of the PF implemented TASSH program. In particular, we will assess the extent to which inner setting variables (e.g., implementation climate, leadership engagement, and organizational capacity) affect the degree of adoption of TASSH at 12 months utilizing structural equation modeling techniques. We will estimate a just-identified path model using the robust weighted least squares estimator to investigate relationships among the theoretical mediators of implementation climate, leadership engagement, and organizational capacity. Based on our conceptual model, we will test the direct effects from the theoretical constructs to adoption. In addition to the direct effects, the indirect effects from the PF intervention to adoption via inner setting variables will be estimated as the product of component direct effects and tested using bootstrapped 95% confidence intervals. As with previous models, random effects will be specified for CHPS and participant to account for the clustered nature of measurements.

#### Consideration of missing data

Although longitudinal designs tend to have missing data due to attrition, we anticipate very little missing data for the adoption ratings as these will come from the staff at the CHPS compounds. For the SBP data, there may be more missing data as these data will be obtained from patients. Using the maximum likelihood multilevel modeling approach to estimate treatment effects, we will include data from all the staff and patients who are enrolled in the trial (intent-to-treat) even if some of the patients’ data are missing. Maximum likelihood estimation, a technique which utilizes observed data to generate parameter estimates for all participants in an intent-to-treat design, has been found to be a useful method for dealing with participant attrition and missing data in clinical trials [42].

#### Data management

A real-time electronic data collection system was established in accordance with stringent data management protocols of Kintampo Health Research Centre (KHRC) and Kwame Nkrumah University of Science and Technology (KNUST). Data will be entered into a web-based electronic database capture system called Research Electronic Data Capture (REDCap) [43]. Electronic data collection forms were developed using the REDCap platform and deployed on tablets for the data collection. Data collection will be done by study coordinators and hosted on a server at KHRC. These forms have in-built data validation and consistency checks. Dedicated study data managers at KHRC will review study data on a weekly basis, generate queries on outliers, and send written queries to the field for correction and updates.

### Oversight and monitoring

*Oversight of study protocol* will be monitored by an independent Data Safety Monitoring Board (DSMB), which will be formed to monitor the safety of the study subjects, and the validity and integrity of the study activities, as well as the data from the study.

*Implementation of the study protocol* will be done by a study team based in Ghana, which comprises the PIs and research coordinators, who will be responsible for the monitoring and quality control of the study. The team meets bi-weekly to monitor the study progress, quality of data collection, adherence to the study manual/protocol, and good clinical practice. Study staff will be trained on all study procedures and documented yearly. The study team will ensure that access to all forms and relevant documents is made available to members of the DSMB upon request, to facilitate their inputs or recommendations on how to improve study activities.

#### Adverse events and serious adverse events

Medical events that occur after enrollment until the end of the study will be classified as adverse events and recorded. Serious adverse events will include life-threatening events and events that may clearly be of major clinical significance, may jeopardize the subject, or may require intervention to prevent one of the other serious outcomes. All adverse events that do not meet any of the criteria for serious [44] will be regarded as non-serious adverse events.

#### Assessment of adverse events

Participants will be evaluated for AEs at each study visit by the physician assistant. Adverse events identified on the field will be reported to the study team immediately. A form will be completed by the study team to capture the relevant information. The study team will report the serious adverse events to all IRBs and to the chair of the DSMB as well as to the sponsor. The DSMB has the authority to halt the implementation if it perceives that harm is occurring due to the intervention. Summaries of adverse events reports will be made available to NIH in the yearly progress report or, at the end of year 5, in the final report, unless the nature of a particular event is such that it bears reporting to NIH immediately. Events will be followed for outcome information until resolution, stabilization, or until the subject completes the study. Participants will be questioned regarding changes in their health and medications. Additionally, the participants’ overall health will be monitored as part of routine standard of care by community health nurse; any changes to the subject’s health will be noted in the subject’s research records.

### Dissemination plan

This trial relies on collaborative work between policymakers and policy implementors within the Ghana Health Service. Thus, during our bi-annual meetings, the research team will present some of the preliminary findings of the study. Also, during the periodic trainings of the community health workers, preliminary findings of the study will be shared with them and the leaders of healthcare delivery for the three contiguous districts. Also, at the community level, the research team and the community health workers will share some of these findings to the community opinion leaders as well as the community members. Additionally, the results will be relevant to other similar settings; hence, the research team will publish the findings in international journals.

## Discussion

Although the systematic uptake of research findings and evidence-based practices into routine practice is a desired outcome of implementation research, there has been little knowledge on “how to do it” empirical literature on the uptake of evidence-based interventions in sub-Saharan Africa [45, 46]. Thus, there is growing interest for researchers to use rigorous research methods to identify factors likely to facilitate or limit the uptake of evidence-based practices in real-world settings. The proposed study is innovative for the following reasons: *First*, it combines well-studied implementation frameworks (i.e., CFIR and RE-AIM) into an intervention to test the effects of inner setting variables and provider-level characteristics on increasing the uptake of evidence-based task-shifting strategies for HTN control. *Second*, it addresses critical gaps in late stage “T4” translation research, in a LMIC with acute health worker shortage [47]. *Third*, it is a multilevel study that utilizes a national policy (CHPS compounds) to address systems-level, provider-level, and patient-level barriers to optimal HTN control. *Fourth*, it engages stakeholders in the research process using a powerful mixed-methods approach designed to consider the needs and priorities of multiple stakeholder groups, who like most stakeholders in SSA are traditionally underrepresented in implementation and dissemination research [45]. *Finally*, our plan to evaluate the uptake of TASSH at the community level is quite innovative given the paucity of data on intervention uptake in community settings in SSA using either a conceptual framework or a structured approach to data collection known for its methodological rigor (i.e., group concept mapping) [48]. This is the only study we are aware of that takes into account the complexity of introducing new evidence-based task-shifting strategies for HTN control into community settings, the barriers to integrating task-shifting into community level care, and the corresponding complex needs of systems, organizations, providers, and patients with preventing, treatment, and control of HTN in adults.

There are several potential methodological challenges that may be encountered during the project. First, it is likely that we will observe variations in the implementation of TASSH and the TSF strategy at CHPS zones randomized to the intervention condition. While we have defined the core elements of the TSF and the TASSH intervention, we acknowledge that adaptation to the unique practice context of CHPS zones will be necessary, particularly given the focus on community-based care. Prior studies suggest that this is an inherent component of practice facilitation with the goal to enhance adoption and sustainability. We will use fidelity checks to engage, enhance, and evaluate tasks of the CHOs so as to ensure that the core elements of the TSF strategy are implemented and documented to enhance external validity. Secondly, unforeseen changes in the outer settings of Ghana Health Systems and the CHPS zones may affect implementation outcomes (i.e., policy changes with HTN control, health insurance scheme). Semi-structured interviews every 6 months on CHO’s delivery of the intervention will be collected at each site to review and analyze for any threats to internal validity. Despite these limitations, the findings have potential for high impact by accelerating the tempo of implementation, adoption, and sustainability of evidence-based task-strengthening strategies for HTN control in sub-Saharan Africa. Findings will also provide policymakers and key stakeholders with the data they need to make decisions regarding dissemination and scale-up of effective task-strengthening strategies for HTN control.

## Trial status

The study-specific manual of operations and policies has been developed. The first cohort (18 CHPS zones) has been randomized and enrolled. Also, 72 CHOs from the 36 zones have been trained to start implementation. The Data Safety and Monitoring Board has been formed and has already had its first meeting which was held on 2 July 2019. Steering Committees have been constituted and already had 4 meetings. The pre-implementation activities and contextual assessment of aim 1 have been completed. Enrolment into the trial started in November 2019 and should be completed by July 2021. This is study protocol version 4.0, and the version date is August 21, 2019.

## Supplementary information

**Additional file 1.** SPIRIT 2013 Checklist: Recommended items to address in a clinical trial protocol and related documents.

## Data Availability

Upon completion of the trial, datasets used and/or analyzed during the current study are available from the corresponding author on reasonable request.

## Abbreviations

BP: Blood pressure
CFIR: Consolidated Framework for Implementation Research
CHOs: Community health officers
CHPS: Community-Based Health Planning and Services
CV: Cardiovascular
CVD: Cardiovascular disease
DBP: Diastolic blood pressure
DSMB: Data Safety Monitoring Board
EBP: Evidence-based practice
EBPAS: Evidence-Based Practice Attitude Scale
HTN: Hypertension
ICR: Identifying, counseling, and referring
IRB: Institutional Review Board
KHRC: Kintampo Health Research Centre
KNUST: Kwame Nkrumah University of Science and Technology
LMIC: Low- and middle-income country
NCDs: Non-communicable diseases
NHLBI: National Heart Lung and Blood Institute
OHRP: Office for Human Research Protections
PF: Practice facilitation
RCT: Randomized controlled trial
REDCap: Research Electronic Data Capture
SAEs: Serious adverse events
SBP: Systolic blood pressure
TASSH: Task-Strengthening Strategy for Hypertension control
TSFs: Task-strengthening facilitators
UC: Usual care
WHO: World Health Organization
